# Assessment of Town and Park Characteristics Related to Physical Activity in the Lower Mississippi Delta

**DOI:** 10.5888/pcd16.180410

**Published:** 2019-03-28

**Authors:** Jessica L. Thomson, Melissa H. Goodman, Alicia S. Landry

**Affiliations:** 1 US Department of Agriculture, Agricultural Research Service, Stoneville, Mississippi; 2 Department of Family and Consumer Sciences, University of Central Arkansas, Conway, Arkansas

## Abstract

**Introduction:**

Our objective was to determine aspects of the built environment that may have contributed to the low levels of physical activity reported in both the gestational and postnatal periods by women participating in a diet and physical activity intervention in the rural Lower Mississippi Delta.

**Methods:**

The built environments of 12 towns were measured by using the Rural Active Living Assessment tools and the Community Park Audit Tool. Correlations between town assessment scores and town size variables were computed by using Kendall τ coefficient. The street distance from a participant’s home address to the nearest park was computed by using network analysis in ArcGIS.

**Results:**

Rural Active Living Assessment scores were low with mean values between 0% (town policy) and 68% (parks and playgrounds) of the highest possible scores. The mean (standard deviation) number of parks per town was 2.6 (3.2), and 55% of the 31 parks were in the 2 largest towns. Most parks (87%) had a single amenity while 1 park had more than 4 amenities. Distance from a participant’s home to the nearest park ranged from less than 0.1 to 8.8 miles (mean [standard deviation], 1.2 [1.8]).

**Conclusion:**

These 12 Lower Mississippi Delta towns scored low on assessments of physical environment features and amenities, town characteristics, and programs and policies associated with physical activity in rural communities. To increase the physical activity levels of rural residents, it may be necessary to first improve the built environment in which they live.

**Summary**

**What is already known on this topic?**

Exercise during and after pregnancy can convey important health benefits for both mother and infant. However, the built environment of rural communities can present challenges for engaging in physical activity.

**What is added by this report?**

The built environments of the Lower Mississippi Delta towns included in this study lacked key programs, policies, and amenities associated with physical activity among residents.

**What are the implications for public health practice?**

Before conducting physical activity interventions in rural communities, it may be necessary to first assess the built environments of target populations.

## Introduction

Lifestyle choices throughout pregnancy can play crucial roles in both the mother’s and her unborn child’s health. Exercise during pregnancy can ease discomforts such as back pain, boost mood and energy levels, improve sleep, prevent excess weight gain, and increase stamina and muscle strength (1–3). Continuing to exercise after giving birth is essential for strengthening and toning abdominal muscles, boosting energy, promoting better sleep, relieving stress, and losing pregnancy weight gain (4,5). Yet less than one-fourth of pregnant women in the United States meet recommendations for physical activity (PA) (6), and women’s participation in exercise programs diminishes after giving birth (1).

From 2013 through 2016, a diet and PA intervention was conducted with pregnant women and their infants residing in the rural Lower Mississippi Delta region of the United States. The Delta Healthy Sprouts (DHS) Project compared the effect of 2 maternal, infant, and early childhood home visiting curricula on health behaviors of women and their infants (7). Analysis of the project’s PA data indicated that baseline PA was low among DHS participants, and positive PA changes were not observed in the gestational or postnatal periods for this cohort of women (8,9). We conducted an observational ancillary investigation, the Delta Neighborhood Physical Activity Study, to determine aspects of these women’s built environment that may have contributed to their low levels of PA.

## Methods

### Study setting

The Delta Neighborhood PA Study included the 12 towns in which DHS participants resided. Parks within these towns were identified 1) by contacting local governing bodies, including city or town hall, mayor’s office, town or county office, parks and recreation department, and park commission office; 2) by conducting internet searches; and 3) by study staff members’ knowledge of the towns. The study was approved and classified as exempt by the Institutional Review Board of Delta State University (IRB protocol number 16–028). Data collection occurred from August 2016 to September 2017.

### Measures

The built environments of the 12 towns were measured by using the Rural Active Living Assessment (RALA) tools: the Program and Policy Assessment (PPA) tool, the Town-Wide Assessment (TWA) tool, and the Street Segment Assessment tool. These 3 observational tools are designed to assess via surveys physical environment features and amenities, town characteristics, and community programs and policies that can affect PA among residents in rural communities (10). Information necessary to complete these surveys was obtained via contact with local governing bodies and school officials, internet searches, staff members’ knowledge of the towns, and direct observation. The PPA tool consisted of 20 questions that provided an inventory of each town’s programs and policies related to PA. Items included policies for bikeways or walkways, presence of a public recreation department or a private organization offering PA programs, local public transportation, school walking programs, sponsored PA for schoolchildren, a late bus option for children participating in after school activities, and the percentage of children living within 1 mile of their school. The TWA tool consisted of questions about 18 town characteristics and an inventory of 14 recreational amenities that measured each town’s physical characteristics on a broad level. Town characteristics included county and town size measures, topography, presence of a town center, street patterns, and location of schools (elementary, middle, high, and magnet). Magnet schools were added to capture the presence of this type of school in some of the towns. The recreational amenity inventory looked for hiking or walking trails, biking paths, public parks, swimming beaches, public use swimming pools, rivers with watersport access, lakes with watersport access, skate parks, ice skating rinks, roller skating rinks, recreational centers, private fitness facilities, playgrounds, and playing fields or courts. We added “lakes with watersport access” to capture the presence of this amenity in one of the towns. The Street Segment Assessment tool consisted of 28 questions that measured each town’s physical characteristics on a detailed (micro) level. Data from the Street Segment Assessment tool is reported elsewhere (11).

Although 2 of the towns exceeded the recommended population size (<10,000) for use of the RALA tools, the surveys were used to assess all of the towns for measurement consistency and comparison among towns. Component and total scores were computed by using scoring algorithms provided in the RALA code and scoring book (10). The higher the assessment scores, the more conducive the town’s built environment was to engagement in PA by its residents.

Because the TWA did not provide a detailed assessment of public parks, the Community Park Audit Tool (CPAT) was used to collect specific information regarding features of the towns’ public parks (12). A public outdoor space with at least 1 identifiable activity area (eg, green space, playground, field, court, walking trail) was used as the operational definition for a public park. School playgrounds were not included in this assessment. The CPAT survey consists of 4 sections: park information (6 items), access and surrounding neighborhood (11 items), park activity areas (15 items), and park quality and safety (16 items). To avoid redundancy, scoring for the TWA parks component was based on data captured with the CPAT because it contained the same information (and more) as that captured with the TWA tool. We used summary measures to present the data captured with the CPAT because no scoring algorithm is available for this instrument.

For RALA training, senior researchers and research associates (data collectors) watched a recorded web-based seminar that discussed the 3 tools. The webinar is available from the Active Living Research team (10). Senior research members reviewed and discussed the RALA codebook with research associates before data collection and verified sources used for obtaining town information after data collection. Training for use of the CPAT consisted of review and discussion of the user guide by senior research members with research associates and field testing of the instrument on 3 parks in a nearby town that was not included in the study catchment. Additionally, we randomly selected 10% of the parks for duplicate measurement by senior research members for quality assurance purposes. Discrepancies between duplicate measurements were discussed and resolved.

We re-created the RALA and CPAT instruments as electronic surveys by using Snap Surveys software (version 11.20, Snap Surveys Ltd). All data were collected via tablets loaded with Snap Surveys software and stored on the Snap WebHost, an online, mobile, and secure survey management system.

### Statistical analyses

Statistical analyses were performed by using SAS (version 9.4, SAS Institute Inc). We considered results significant at the nominal level of *P* < .05. Kendall τ coefficient, a nonparametric measure of an association’s strength and direction, was used to compute correlations between town assessment scores and town size variables because most of the distributions were highly skewed. Analyses of the RALA data sets were conducted both with and without the 2 towns that exceeded the recommended population size (<10,000) for use of the RALA tools. We used the longitude and latitude coordinates of each park’s center to mark its location. The street path distance from a participant’s home address to the nearest park was computed by using network analysis in ArcGIS (version 10.4, Esri). Three of the 12 towns did not contain any parks. For 2 of the 4 participants living in these 3 towns, their nearest park was in a measured town. For the other 2 participants, their nearest park was in neighboring towns that were not measured because no participants lived in these towns. Although the 2 parks in the nonmeasured towns were used for computing distance to the nearest park, these 2 parks were not measured because we focused on the towns in which participants lived.

## Results

### Rural Active Living Assessment

Most (63%) DHS participants lived within the boundaries of the 2 largest towns in the intervention. At baseline, none of the 82 DHS participants met the recommended 150 minutes per week of moderate intensity PA (13). However, 5 participants were classified as engaging in moderate amounts of PA, while the other 77 were classified as engaging in low amounts (13). Four of the 5 participants who engaged in moderate amounts of PA lived in the largest town while the fifth participant lived in the third largest town. Mean town population size was 5,319, and mean density was 1,280 residents per square mile (Table 1). Town PPA component and total scores were low, with mean values between 0% (town policy) and 50% (school policy) of the highest possible scores on the assessment. Town TWA component and total scores also were low, with mean values between 19% (amenity) and 68% (parks and playgrounds) of the highest possible scores on the assessment. Mean scores were lower when the 2 largest towns were excluded from the analyses, with the exception of the town policy score (zero for all towns).

**Table 1:** Characteristics of Towns in the Delta Neighborhood Physical Activity Study, 2016–2017

Characteristic	Range of Possible Scores	All Towns (n = 12)				Largest Towns^a^ Excluded (n = 10)		
		Mean (SD)	Median	Min^b^	Max	Mean (SD)	Median	Max
**Population^c^**	NA	5,319 (9,739)	1,743	337	34,400	1,709 (1,419)	1,321	4,481
**Area (square miles)^c^**		4 (7)	1	1	27	2 (1)	1	4
**Density (per square mile)^c^**		1,280 (701)	1,262	461	2,519	1,188 (706)	1,178	2,519
**Program and Policy Assessment**								
Town program score^d^^,^ e	0–30	6 (9)	0	0	26	3 (6)	0	14
Town policy score^d^^,^ f	0–10	0 (0)	0	0	0	0 (0)	0	0
School program score^d^^,^ g	0–30	5 (9)	0	0	30	3 (5)	0	10
School policy score^d^^,^ h	0–30	15 (13)	15	0	30	14 (13)	15	30
Total score^d^^,^ i	0–100	26 (25)	29	0	86	19 (18)	22	40
**Town-Wide Assessment**								
School count	NA	5 (7)	3	0	23	3 (3)	3	9
School score^d^^,^ j	0–21	6 (6)	5	0	15	5 (6)	2	15
Amenity type count^d^^,^ k	NA	4 (2)	4	0	8	3 (2)	3	5
Amenity total count^d^^,^ l	NA	10 (11)	6	0	37	5 (5)	4	12
Amenity score^d^^,^ m	0–53	10 (9)	9	0	29	7 (6)	8	18
Parks and playgrounds score^d^^,^ n	0–25	17 (11)	23	0	25	15 (11)	19	25
Total score^d^^,^ o	0–99	32 (21)	36	0	62	27 (18)	29	52

Abbreviations: Min, minimum; Max, maximum; NA, not applicable; SD, standard deviation.

a Excluded 2 towns with populations exceeding recommended size (<10,000) for Rural Active Living Assessment tools.

b Minimum values are the same for both sets of towns.

c Source: www.factfinder.census.gov.

d Higher scores indicate the town’s built environment was more conducive to physical activity.

e Composed of 6 items concerning public and private recreation.

f Composed of 1 item concerning bikeways/walkways required for new infrastructure.

g Composed of 2 items concerning public access to recreation facilities and late bus options.

h Composed of 3 items concerning walking and safe routes to school and sponsored physical activity programs.

i Sum of scores for town program, town policy, school program, and school policy.

j Composed of 4 items concerning walkability to schools (elementary, middle, high, and magnet).

k Count of different types of amenities (each of 17 types counted only once).

l Count of total number of amenities (may include multiples of same type).

m Composed of 13 items concerning location of amenities from town centers.

n Composed of 4 items concerning location of parks, playgrounds, and sports fields and courts from town centers.

o Sum of scores for school, amenity, and parks and playgrounds.

With all towns included, town population was significantly associated with the PPA total score and its school policy component score (Table 2). Town population also was significantly associated with the TWA total score and its school and parks and playgrounds components scores. Town population density was significantly associated with the PPA total score and its school program and school policy component scores as well as the TWA school component score. All correlations were in the positive direction indicating that as town size increased, assessment scores also increased. Town area (square miles) was not significantly associated with any of assessment scores when all towns were included in the analyses. Associations generally increased in magnitude when the 2 largest towns were excluded from the analyses.

**Table 2:** Associations Among Town Size Measures and Rural Active Living Assessment Scores, Delta Neighborhood Physical Activity Study, 2016–2017

Town Size	Statistic	Program and Policy Assessment Scores^a^				Town-Wide Assessment Scores			
		Town Program	School Program	School Policy	Total	School	Amenity	Parks and Playgrounds	Total
**All towns included (n = 12)**									
Population	KTC	0.30	0.49	0.54	0.58	0.77	0.32	0.76	0.72
	*P* value	.30	.09	.04	.03	.004	.22	.004	.004
Area (square miles)	KTC	0.31	0.00	0.07	0.11	0.36	0.07	0.51	0.35
	*P* value	.33	>.99	.81	.72	.23	.82	.08	.22
Population density (per square mile)	KTC	0.00	0.62	0.60	0.63	0.56	0.22	0.37	0.45
	*P* value	>.99	.03	.03	.02	.03	.40	.16	.07
**Largest towns^b^ excluded (n = 10)**									
Population	KTC	0.56	0.48	0.53	0.60	0.68	0.55	0.79	0.78
	*P* value	.02	.05	.03	.01	.004	.02	.001	<.001
Area (square miles)	KTC	0.64	0.20	0.24	0.33	0.42	0.46	0.64	0.56
	*P* value	.02	.46	.37	.20	.10	.07	.01	.02
Population density (per square mile)	KTC	0.15	0.40	0.53	0.50	0.61	0.29	0.39	0.47
	*P* value	.56	.11	.03	.03	.009	.21	.10	.03

Abbreviation: KTC, Kendall τ correlation.

a Town policy was not included since all towns scored 0 points on this component.

b Excluded 2 towns with populations exceeding recommended size (<10,000) for Rural Active Living Assessment.

### Community parks audit

All 31 parks were measured on a weekday in the fall of 2016. Three of the 12 towns did not have any parks, 4 towns had a single park, 2 towns had 3 parks, and the remaining 3 towns had 4, 6, and 11 parks. The mean number (standard deviation [SD]) of parks per town was 2.6 (3.2) and over half of the parks (n = 17) were in the 2 largest towns. Most parks were easy to find (25 [81%]), accessible (29 [94%]), and had at least 6 entry points or an open boundary (18 [58%]) (Table 3). Of the 7 neighborhood concerns observed, the most frequent was no or low street lighting (26%), followed by graffiti (13%) and poorly maintained property (13%). For 14 (45%) of the parks, no neighborhood concerns were observed. A smaller number of concerns were observed for the parks themselves including graffiti (26%), excessive litter (13%), and poor maintenance (7%). In 19 (61%) of the parks, no park concerns were observed. In terms of aesthetics, almost all (94%) of the parks had scattered trees present, although only 8 (26%) parks featured landscaping (eg, flower beds, pruned bushes).

**Table 3:** Characteristics of Parks (N = 31) Included in the Delta Neighborhood Physical Activity Study, 2016–2017

Characteristic	n (%)
**Park Characteristics**	
**Easy to find**	25 (80.6)
**Accessible**	29 (93.5)
**Points of entry**	
1	3 (9.7)
2–5	10 (32.3)
≥6 or open boundary	18 (58.1)
**Parking type**	
Lot	18 (58.1)
On street	14 (45.2)
None	1 (3.2)
**Bordering sidewalks^a^**	8 (25.8)
**Bordering traffic signs^b^**	25 (80.6)
**Main land use**	
Residential	22 (71.0)
Institutional (school)	1 (3.2)
Commercial	4 (12.9)
Natural	4 (12.9)
**Neighborhood concerns^c^**	
No or low street lighting	8 (25.8)
Graffiti	4 (12.9)
Poorly maintained property	4 (12.9)
Excessive litter	3 (9.7)
Heavy traffic	3 (9.7)
Vacant/abandoned buildings	2 (6.5)
Unfavorable buildings	2 (6.5)
None	14 (45.2)
**Park concerns^d^**	
Graffiti	8 (25.8)
Excessive litter	4 (12.9)
Poor maintenance	2 (6.5)
None	19 (61.3)
**Aesthetic features**	
Scattered trees	29 (93.5)
Dense trees	10 (32.3)
Landscaping	8 (25.8)
Water	6 (19.4)
Historical/educational	4 (12.9)
**Activity Area Characteristics**	
**Total number**	
1	27 (87.1)
2	1 (3.2)
3	2 (6.5)
≥4	1 (3.2)
**Open or green space**	27 (87.1)
**Playground^e^**	24 (77.4)
Good condition	22 (88.0)
Colorful equipment	22 (88.0)
Shade cover ≥25%	11 (44.0)
Bench	18 (72.0)
Separation from road	11 (44.0)
**Basketball court**	16 (51.6)
Good condition	13 (81.3)
**Baseball field^f^**	12 (38.7)
Good condition	14 (100.0)
**Trail^g^**	8 (25.8)
Good condition	9 (100.0)
Connected to activity areas	8 (88.9)
Bench for sitting	4 (44.4)
**Sport field^h^**	4 (12.9)
Good condition	5 (100.0)
**Tennis court**	4 (12.9)
Good condition	4 (100.0)
**Swimming pool**	3 (9.7)
Good condition	2 (66.7)
**Volleyball court**	3 (9.7)
Good condition	2 (66.7)
**Fitness equipment or station**	2 (6.5)
Good condition	2 (100.0)
**Feature Characteristics**	
**Lights**	27 (87.1)
**Trash can**	26 (83.9)
Overflowing	0 (0.0)
**Restroom^i^**	14 (45.2)
Good condition	12 (85.7)
**Bench for sitting**	21 (67.7)
Good condition	19 (90.5)
**Drinking fountain**	6 (19.4)
Good condition	2 (33.3)
**Picnic table**	14 (45.2)
Good condition	14 (100.0)
**Picnic shelter**	13 (41.9)
**Grill/fire pit**	11 (35.5)
**Animal rules posted**	11 (35.5)
**Interior road**	4 (12.9)
**Recycling container**	1 (3.2)
**Vending machine**	1 (3.2)

a All sidewalks were useable, but only 5 of the 8 parks had curb cuts or ramps.

b 24 parks had stop signs, 1 park had a stop light, and no parks had crosswalks.

c None of the surrounding neighborhoods had vandalism, excessive noise, lack of eyes on the street, or threatening persons or behavior.

d None of the parks had vandalism, excessive noise or animal waste, threatening persons or behavior, or dangerous spots.

e 24 parks had playgrounds, but 1 park had 2 playground areas so denominator is 25 for playground features.

f 12 parks had baseball fields, but 2 parks had 2 baseball fields so denominator is 14 for field condition.

g 8 parks had trails, but 1 park had 2 trails so denominator is 9 for trail features.

h Football or soccer fields; 4 parks had sports fields, but 1 park had 2 sport fields so denominator is 5 for field condition.

i Included portable toilets.

Most parks (87%) had a single activity amenity while 1 park had more than 4 amenities. The most common amenities were open or green spaces (87%) followed by playgrounds (77%) and basketball courts (52%). Amenities were in good condition, ranging from 67% for volleyball courts and swimming pools to 100% for baseball fields, trails, sports fields, tennis courts, and fitness equipment or stations. In terms of features, most parks had lights (87%), trash cans (84%), and benches for sitting (68%). Less than half the parks had restrooms (45%), picnic tables (45%), picnic shelters (42%), grills or fire pits (36%), or drinking fountains (19%). In all 31 parks, the following features were absent: map of the park, public transit stop near park, bike racks, bike routes bordering park, splash pads, off-leash dog parks, family restrooms, and baby changing stations in the restrooms.

Street path distance from a participant’s home to the nearest park ranged from less than 0.1 to 8.8 miles (mean [SD], 1.2 [1.8]). For the 5 participants who engaged in moderate amounts of PA at baseline, mean (SD) distance from home to the nearest park was 1.0 (1.3) miles, compared with 1.2 (1.8) miles for the participants who engaged in low amounts of PA at baseline. Additionally, 3 of the 5 participants who engaged in moderate amounts of PA lived close (one-half mile or less) to a park. In comparison, 47% of participants who engaged in low amounts of PA lived within one-half mile of a park.

## Discussion

We presented physical activity–related characteristics of the towns in which DHS participants lived as well as features and amenities of the parks in these towns. Results indicate that the built environment may have played a role in the low levels of PA observed in this cohort of rural, Southern, primarily African American women. On average, assessment scores for programs, policies, features, and amenities related to PA were low for the towns in which these women lived. Mean PPA and TWA scores for towns in our study (26 and 32) were lower than those reported in studies assessing rural towns in the Deep South (55 and 59), Appalachia region of North Carolina (not assessed and 50), Washington Latino communities (69 and 63), and Hawaii (39 and 67) (14–17). Similar to findings in our study, all but 1 Deep South community scored zero on the PPA town policy (14). However, all towns in the previous 4 studies had recreational amenities (14–17), while 2 towns in our study had no recreational amenities. Furthermore, no town in our study had public transportation systems. Results from a systematic review of correlates of PA suggest that recreational facilities must be present and either close to an person’s residence with safe walking routes or accessible by public transportation to promote participation in PA at such facilities (18).

In our study, towns with higher populations had higher TWA total and parks and playgrounds component scores, indicating that larger towns were more conducive to engagement in PA by their residents. A similar relationship between town size and TWA scores was present in the Washington towns, but not in the Deep South, North Carolina Appalachian, or Hawaiian towns (14–17). In our study, 4 of the 5 participants who engaged in moderate amounts of PA at baseline lived in the most populated town with the highest parks and playgrounds component score (25) and the second highest TWA score (60). The other participant lived in the third most populated town also with the highest parks and playgrounds component score (25) and the third highest TWA score (52). Results should be interpreted cautiously because of the small number of participants who engaged in moderate amounts of PA.

Almost half of DHS participants lived within one-half mile walking distance of a park and approximately three-fourths of the parks contained playgrounds, an amenity associated with park-based PA in women (19). However, only one-fourth of the parks had bordering sidewalks, which suggests that walking routes to parks lacked this safety feature. In a telephone survey conducted with 1,176 South Carolina residents, more African American women reported greater maintenance of sidewalks as a correlate of PA than white women did (20). Likewise, the presence of sidewalks and feeling safe and secure from crime and traffic were closely linked to the decision to be physically active in minority women (21). Hence, walking routes to and around public parks may have been a contributing factor to the low levels of PA observed among DHS participants. Potentially compounding the issue of safe walking routes is aesthetics, which also was identified as an important environmental design aspect in the systematic review of correlates and determinants of PA in adults and children (18). In our study, all but 2 of the 31 parks had scattered trees present, but only 8 featured landscaping. Thus, the lack of aesthetic features in most of the parks may have at least partly discouraged engaging in PA in the parks in this cohort of women.

The built environment likely played a role in the low levels of PA observed in these women; however, the influence of their personal health characteristics bears mentioning. During pregnancy, PA levels are known to decrease (22), probably because of anatomic and physiologic changes that occur. Additionally, two-thirds of the women in our study were overweight or obese before becoming pregnant and scored relatively low for PA self-efficacy at baseline (13). Overweight classification and lower self-efficacy for participating in PA have been negatively related to PA levels (18).

Strengths of this study are the use of multiple validated and objective tools to assess town and park characteristics and exploration of potential associations between study participants’ PA levels with town and park measures and features. The population studied also is a strength because rural, Southern, African American adults are at increased risk for inadequate amounts of PA (23–25). A limitation is the small sample sizes for both study participants and towns, which may have limited the ability to find significant associations in the data. Additionally, the nonrandom selection of towns and parks limits the generalizability of the study’s results.

The Lower Mississippi Delta towns included in this study generally scored low on assessments of physical environment features and amenities, town characteristics, and community programs and policies that can affect PA among residents in rural communities. Furthermore, although most DHS participants lived close to a park, the parks lacked features known to be associated with PA, such as safe walking routes and aesthetics. To increase PA levels of rural residents, it may be necessary to first improve the built environment in which they live.
